# De novo design of potent and resilient hACE2 decoys to neutralize SARS-CoV-2

**DOI:** 10.1126/science.abe0075

**Published:** 2020-11-05

**Authors:** Thomas W. Linsky, Renan Vergara, Nuria Codina, Jorgen W. Nelson, Matthew J. Walker, Wen Su, Christopher O. Barnes, Tien-Ying Hsiang, Katharina Esser-Nobis, Kevin Yu, Z. Beau Reneer, Yixuan J. Hou, Tanu Priya, Masaya Mitsumoto, Avery Pong, Uland Y. Lau, Marsha L. Mason, Jerry Chen, Alex Chen, Tania Berrocal, Hong Peng, Nicole S. Clairmont, Javier Castellanos, Yu-Ru Lin, Anna Josephson-Day, Ralph S. Baric, Deborah H. Fuller, Carl D. Walkey, Ted M. Ross, Ryan Swanson, Pamela J. Bjorkman, Michael Gale, Luis M. Blancas-Mejia, Hui-Ling Yen, Daniel-Adriano Silva

**Affiliations:** 1 Neoleukin Therapeutics Inc., Seattle, WA, USA.; 2 School of Public Health, Li Ka Shing Faculty of Medicine, University of Hong Kong, Hong Kong Special Administrative Region, China.; 3 Division of Biology and Biological Engineering, California Institute of Technology, Pasadena, CA, USA.; 4 Center for Innate Immunity and Immune Disease, Department of Immunology, University of Washington, Seattle, WA, USA.; 5 Center for Vaccines and Immunology, University of Georgia, Athens, GA, USA.; 6 Department of Epidemiology, University of North Carolina at Chapel Hill, Chapel Hill, NC, USA.; 7 Department of Microbiology, University of Washington, Seattle, WA, USA.; 8 Department of Infectious Diseases, University of Georgia, Athens, GA, USA.

## Abstract

We developed a de novo protein design strategy to swiftly engineer decoys for neutralizing pathogens that exploit extracellular host proteins to infect the cell. Our pipeline allowed the design, validation, and optimization of de novo human angiotensin-converting enzyme 2 (hACE2) decoys to neutralize severe acute respiratory syndrome coronavirus 2 (SARS-CoV-2). The best monovalent decoy, CTC-445.2, bound with low nanomolar affinity and high specificity to the receptor-binding domain (RBD) of the spike protein. Cryo–electron microscopy (cryo-EM) showed that the design is accurate and can simultaneously bind to all three RBDs of a single spike protein. Because the decoy replicates the spike protein target interface in hACE2, it is intrinsically resilient to viral mutational escape. A bivalent decoy, CTC-445.2d, showed ~10-fold improvement in binding. CTC-445.2d potently neutralized SARS-CoV-2 infection of cells in vitro, and a single intranasal prophylactic dose of decoy protected Syrian hamsters from a subsequent lethal SARS-CoV-2 challenge.

## A decoy to neutralize SARS-CoV-2

Many efforts to develop therapies against severe acute respiratory syndrome coronavirus 2 (SARS-CoV-2) are focused on the interaction between the spike protein, which decorates the surface of the virus, and its host receptor, human angiotensin-converting enzyme 2 (hACE2). Linsky *et al.* describe a de novo design strategy that allowed them to engineer decoy proteins that bind to the spike protein by replicating the hACE2 interface. The best decoy, CTC-445, bound with low nanomolar affinity, and selection of viral mutants that decrease binding is unlikely because this would also affect binding to hACE2. A bivalent version of CTC-445 bound even more tightly, neutralized SARS-CoV-2 infection of cells, and protected hamsters from a SARS-CoV-2 challenge. The stable decoy has the potential for respiratory therapeutic delivery.

*Science*, this issue p. 1208

## Teaser

Designed de novo protein decoys neutralize SARS-CoV-2 in vitro and in vivo and are resilient to viral mutational escape.

Since its emergence as a global pandemic in December of 2019, severe acute respiratory syndrome coronavirus 2 (SARS-CoV-2) has caused millions of COVID-19 cases. The need for effective strategies to prevent and treat the disease remains urgent (*1*). There are multiple ongoing efforts to develop prophylactics and therapeutics using various approaches (*2*) such as vaccination (*3*), traditional protein engineering (*1*, *4*, *5*), de novo protein design (*6*), and small-molecule drug discovery (*7*). A challenge is that the high mutational rate of positive sense single-strand RNA (+ssRNA) viruses (*8*–*10*) can often lead to viral escape (*11*), which could compromise the efficacy of many SARS-CoV-2 therapeutics under development. Several mutations have already occurred in the S protein of SARS-CoV-2 in the infected population (*12*, *13*). Deep-sequencing studies of the receptor-binding domain (RBD) have shown that simple mutations can enable the virus to escape known netralizing antibodies or to increase its binding affinity for human angiotensin-converting enzyme 2 (hACE2) (*14*, *15*), the membrane protein that the virus exploits to gain entry into the cell. There is thus a pressing need to develop new therapeutics that can be more resistant to SARS-CoV-2 mutational escape.

Traditional approaches to combatting viruses (e.g., vaccination and monoclonal antibodies) rely on molecules interacting with the pathogens in a way that is fundamentally different from how the pathogen engages with its cellular targets (*16*, *17*). Viruses can be selected to evade neutralization, undergoing protein mutations that prevent recognition by the neutralizing molecules (e.g., antibodies) while preserving viral fitness. To address these challenges, we have developed a computational protein design strategy that enables the rapid and accurate design of hyperstable de novo protein “decoys” that replicate the protein receptor interface to which a virus binds to infect a cell. The decoys can achieve a similar or even higher affinity than the original protein receptor by stabilizing the binding interface. Therefore, at an optimal concentration, the decoys can outcompete viral interaction with the cell.

**Fig. 1:**
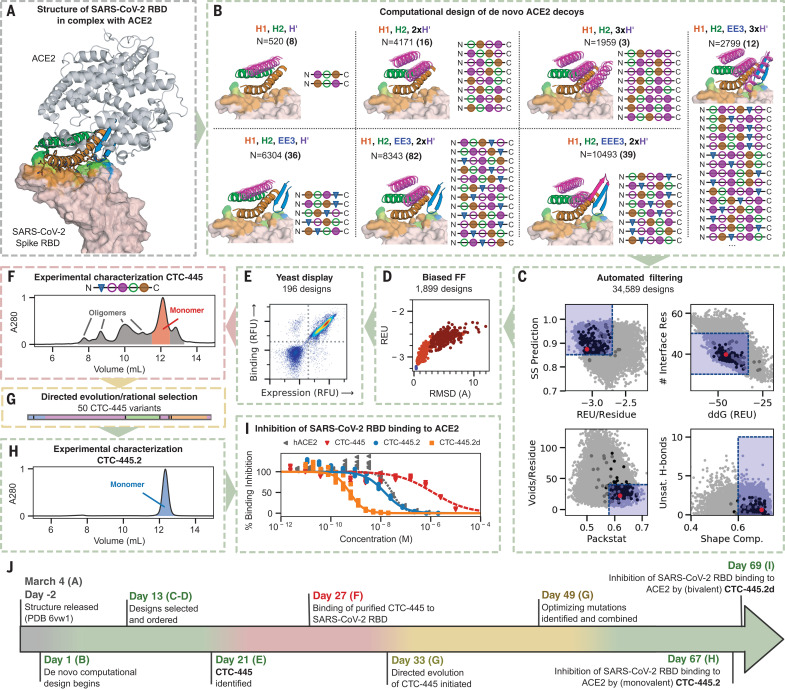
Design and characterization of de novo ACE2 decoys. (**A**) ACE2 (gray) and its binding motifs (H1 19-52, orange; H2 55-84, green; EE3 346-360, blue) in complex with SARS-CoV-2 RBD (pink). Three starting structures were simultaneously used as targets (see main text); 6VW1 is shown. (**B**) De novo secondary structure elements (magenta) were computationally generated to stabilize H1, H2, and EE3. Seven combinations of secondary structure elements were considered. Circles are α-helices, triangles are β-sheets, filled circles are helices oriented forward, and empty circles are helices oriented backward. We used Rosetta to generate fully connected backbones (using the “protein_mimic_designer” algorithm) and amino acid sequences predicted to fold into the target structure. In all cases, the binding interface of ACE2 with the SARS-CoV-2 RBD was preserved intact (see the materials and methods). (**C**) Automatic computational filtering based on eight metrics selected the best candidates. The RMSD of the binding motifs to ACE2 was also used as a quality check. The dots indicate the mean computational score for each design scored against the three target RBD structures. Designs selected for experimental testing are shown in black. Our best design, CTC-445, is shown in red. The blue boxes indicate the filtering thresholds (see the materials and methods). (**D**) Designs that passed filtering were subjected to biased forward folding simulations (see the materials and methods), here shown for CTC-445, including the unsalted biased simulation (brown), the native-salted simulation (orange), and relaxation (blue). (**E**) The top 196 designs were selected for yeast display screening using a combination of Rosetta score per residue, the ddG Rosetta filter, and the folding simulations (see the materials and methods). The designs were individually assessed for specific binding to SARS-CoV-2 spike RBD (Fc fusion, 200 nM). The plot for CTC-445 is shown. (**F**) CTC-445 was recombinantly expressed and purified by affinity chromatography (see the materials and methods). Analytical size exclusion chromatography (SEC) for CTC-445 revealed the presence of oligomeric species. (**G** and **H**) CTC-445 was optimized by directed evolution and rational combination of the observed favorable mutations (G), leading to CTC-445.2 (SEC), which is mainly monomeric in solution (H) and ~1000× more potent to compete with ACE2 than its parent [see (G)]. We further optimized the potency of our molecule by generating a bivalent version named CTC-445.2d. (**I**) Potency of designs to outcompete binding of SARS-CoV-2 RBD to ACE2, as measured by competition enzyme-linked immunosorbent assay (ELISA) using a constant concentration of 0.4 nM ACE2. (**J**) Timeline of the de novo protein design and optimization pipeline. Timewise, green indicates phases that we believe were performed optimally, red indicates those that can potentially be avoided in future efforts, and yellow indicates phases that can potentially be expedited by using more advanced and/or automated methods for gene synthesis, cloning, and high-throughput screening.

SARS-CoV-2 invades host cells in a two-step process (*18*–*20*). The S protein RBD attaches to the cell by binding to hACE2, a membrane-associated protein, triggering protease-mediated fusion with the cell membrane (*21*). The process is similar to the beta-coronaviruses HCoV-NL63 and SARS-CoV-1, which also target hACE2 for cellular entry (*22*). In principle, inhibiting the viral interaction with hACE2 should prevent infection. We applied our design strategy to engineer, validate, and optimize de novo hACE2 decoys to neutralize SARS-CoV-2 infection (Fig. 1J and fig. S1). The design of the decoys started by identifying the structural motifs that form the hACE2 binding interface with the SARS-CoV-2 RBD. We based our effort on three publicly available structures of hACE2 in complex with the RDB of the S protein for SARS-CoV-1 (PDB: 6CS2) and SARS-CoV-2 (PDBs: 6VW1 and 6M17) (*23*–*25*). Four discontiguous binding elements were identified (Fig. 1A) and the three largest interacting motifs were selected to build the de novo decoys: two long alpha helices (H1 and H2) and a short beta hairpin (EE3) (Fig. 1A and fig. S2). To generate molecules that are biologically inert for humans, our computational design strategy avoided incorporating elements of hACE2 that are known (or predicted) to be biologically active, such as the catalytic site. Inspired by recent developments in the design of de novo structural elements (*26*–*29*), we built new disembodied de novo secondary structure elements tailored to support the target structural elements in a way that is both compatible with globular folding and would stabilize the binding interface (Fig. 1B and materials and methods). Then, in a strategy similar to the design of Neoleukin-2/15 (Neo-2/15) (*26*, *30*), a combinatorial design approach based on Rosetta’s “protein_mimic_designer” was used to generate multiple fully connected protein topologies containing all of the desired structural and binding elements (*26*). The design of the protein decoys was constrained to fully preserve (intact up to each amino acid’s conformation) the target binding interface (Fig. 1, A and B, and fig. S2) so that the de novo proteins would be resilient to viral mutational escape. Rosetta (*31*) was then used to generate amino acid sequences predicted to fold into the target structures, and the designs were evaluated with an automatic filtering pipeline based on nine computational parameters, including predictions of smooth folding funnels into a stable native state (Fig. 1, C and D) (*32*).

**Fig. 2:**
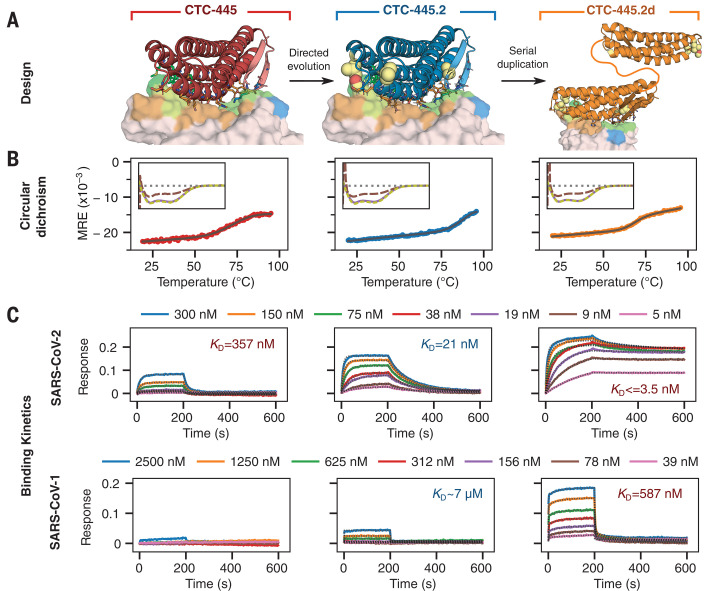
Stability and binding of the de novo protein decoys CTC-445, CTC-445.2, and CTC-445.2d. (**A**) Design models of CTC-445, CTC-445.2, and CTC-445.2d. CTC-445.2 contains five mutations that were guided by directed evolution experiments. CTC-445.2d is a bivalent variant composed of two CTC-445.2 subunits linked by a 16-mer flexible GS linker (sequence -GGGSGGSGSGGSGGGS-). (**B**) Circular dichroism of recombinantly expressed CTC-445 (red), CTC-445.2 (blue), and CTC-445.2d (orange). Thermally induced melting of the decoys was followed by its circular dichroism signal at 208 nm (heating rate, 2°C/min). The inset shows far ultraviolet (UV) wavelength spectra at 20°C (purple), after heating to ~95°C (brown), and after cooling the heated sample to 20°C (green dashed). Complete ellipticity spectra recovery (full reversibility) upon cooling was observed in all cases. Calculated *T*_m_ values for CTC-445, CTC-445.2, and CTC-445.2d are 75.3 ± 0.2°C, ≈93°C, and 71.7.± 0.2°C, respectively. (**C**) Binding was assessed using biolayer interferometry (OCTET) binding assays of CTC-445, CTC-445.2, and CTC-445.2d against immobilized SARS-CoV-2 RBD (top) or SARS-CoV-1 RBD (bottom) (see table S1). The model fitting is shown with dotted black lines.

Approximately 35,000 computational ACE2 decoys were generated, and the top-ranking 196 designs (see the materials and methods) were selected for experimental testing for binding to SARS-CoV-2 RBD using yeast display (Fig. 1E). With no further optimization, the design CTC-445 showed strong (nanomolar) and specific binding for SARS-CoV-2 RBD (Fig. 1E, fig. S3, and materials and methods). CTC-445 is a 160–amino acid protein comprising 18 of the natural amino acids; it does not contain cysteine or tryptophan residues. It exhibited ~10-fold weaker binding affinity for SARS-CoV-2 than did hACE2 [disassociation constant (*K*_D_) ~ 357 nM, *K*_D_ ~ 31 nM, respectively; table S1] and, as a result, CTC-445 was a weak competitor of SARS-CoV-2 RBD binding to hACE2 [median inhibitory concentration (IC_50 @ hACE2[0.4nM]_) = 1.7 μM; Fig. 1I). We determined that low potency of CTC-445 was due to a certain degree of instability of its folded state [free energy difference between folded and intermediate states (Δ*G*_NI_) ~–2.7 kcal mol^−1^, melting transition temperature (*T*_m_) ~75.3°C; Figs. 1F and 2B and fig. S5]. A single round of directed evolution to improve stability and binding affinity, and subsequently the rational combination of the five most frequent observed mutations (none of them in the binding interface), led to the protein decoy CTC-445.2 (Fig. 1G, figs. S6 and S7, table S2, and materials and methods). CTC-445.2 is predominantly monomeric (Fig. 1H and fig. S8), thermodynamically hyperstable (Δ*G*_NI_ ~–5.0 kcal mol^−1^, *T*_m_ ~93°C; Fig. 2B and fig. S5), exhibits low nanomolar affinity for the RBD of SARS-CoV-2 (*K*_D_ ~21.0 nM; table S1), has improved cross-reactivity to SARS-CoV-1 (*K*_D_ ~7.1 μM; Fig. 2C and table S1), and can efficiently compete hACE2 binding to the SARS-CoV-2 RBD (IC_50 @ hACE2[0.4nM]_ ~10.4 nM; Fig. 1I). The amino acid sequence of CTC-445.2 has little identity with hACE2, in terms of either linear or structurally aligned sequence (ClustalW ~22%, MICAN ~ 4%, respectively; fig. S9). Serial duplication (i.e., increase in avidity) of CTC-445.2 led to higher-potency molecules with favorable biochemical properties. For example, CTC-445.2d (Fig. 2A), a bivalent version of CTC-445.2, had an ~10-fold improvement in binding affinity for both SARS-CoV-2 RBD (*K*_D_ ~3.5 nM; table S1) and SARS-CoV-1 RBD (*K*_D_ ~587 nM; Fig. 2C and table S1), and a similar increase in its ability to compete with hACE2 binding to SARS-CoV-2 RBD (IC_50 @ hACE2[0.4nM]_ ~700 pM; Fig. 1I). A trivalent version of CTC-445.2 resulted in even higher (picomolar) binding affinity and a matching hACE2 competition potency (*K*_D_ ~270 pM, IC_50 @ hACE2[0.4nM]_ ~10 pM; fig. S10 and table S1). In a cross-reactivity binding assay containing >21,000 human proteins, we confirmed that CTC-445.2d bound to the SARS-CoV-2 RBD with high selectivity (fig. S11 and materials and methods).

**Fig. 3:**
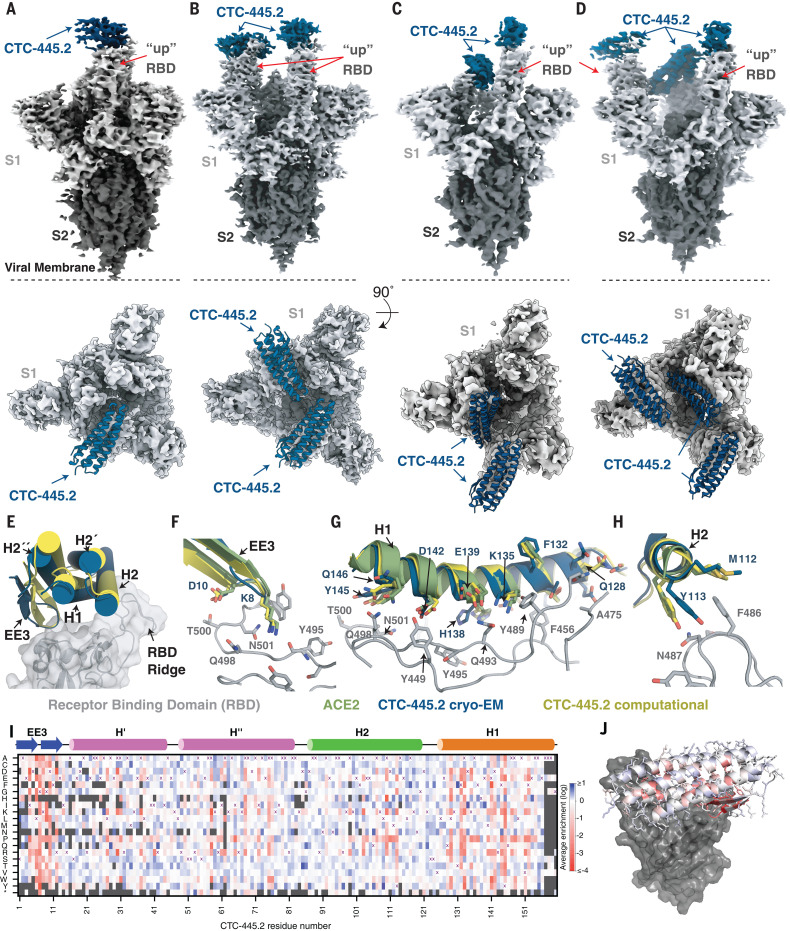
Cryo-EM structure of the CTC-445.2–S complex. (**A** to **D**) Cryo-EM reconstructions of CTC-445.2 (blue) bound to soluble spike trimers (gray). 3D classification revealed four distinct classes: one CTC-445.2 bound to an “up” RBD (A), two CTC-445.2 bound to two “up” RBDs (B), two CTC-445.2 bound to one “up” and one “down” RBD (C), and three CTC-445.2 bound to two “up” and one “down” RBD (D). (**E**) Overlay of CTC-445.2-RBD computationally modeled (yellow) and experimentally determined using cryo-EM (blue). The Cα RMSD between the design model and the refined experimental structure is 1.1 Å. (**F** to **H**) Comparison of cryo-EM CTC-445.2 (blue), computationally modeled CTC-445.2 (yellow), and hACE2 (green) at the interface of the RBD (gray). (**I**) Deep mutational scanning heatmap showing the average effect on the enrichment for single site mutants of CTC-445.2 when assayed by yeast display for binding to the SARS-CoV-2 RBD (binding assayed at RBD concentrations of 100, 50, 25, 12.5, 6.25, 3.125, and 1.5625 pM; see the materials and methods). (**J**) Design model of CTC-445.2 colored by average enrichment at each residue position [from the data in (I)] bound to SARS-CoV-2 RBD (gray). As expected, mutations in the core of the design or to positions involved in binding to the RBD are generally disallowed. The deep mutational scanning revealed that there is still room to further improve the binding affinity of CTC-445.2, including mutations in the binding interface that in principle could afford higher potency and selectivity at the cost of compromising the decoy’s mutational escape resiliency (see Fig. 4).

Single-particle cryo-EM structures of CTC-445.2 in complex with the SARS-CoV-2 S trimer showed that the de novo decoy is capable of simultaneous binding to all three RBDs of the SARS-CoV-2 trimeric S protein, both in the “up” and “partially down” RBD conformations (Fig. 3, A to D, and fig. S12). To accurately model the CTC-445.2-RBD interactions, we used focused classification and local refinement on the subset of particles that showed CTC-445.2 bound to a partially down RBD, which yielded a 4.1-Å map with improved CTC-RBD features relative to CTC-RBD regions on the up RBDs (Fig. 3, A to D, and figs. S12 and S13). The computationally derived model of CTC-445.2 closely matched the cryo-EM–determined structure [Cα root mean square deviation (RMSD) = 1.1 Å], with minor differences observed in the N-terminal EE3 and H2 helix (Fig. 3, E to H). As designed, the binding interface of the SARS-CoV-2 RBD with CTC-445.2 closely mirrored the target hACE2 interface. We used site saturation mutagenesis (SSM; see the materials and methods) (*33*, *34*) to explore the effect of single–amino acid substitutions in CTC-445.2 on its binding to the SARS-CoV-2 RBD (Fig. 3, I and J). The experiment showed that mutations in the core of the design are disallowed, and mutations in surface or exposed residues are generally tolerated (Fig. 3, I and J). The SSM experiment also revealed that there is room to further improve the affinity of the protein by introducing mutations in the binding interface (Fig. 3I), although doing so would break the hACE2 structural mirroring of the de novo decoy.

**Fig. 4:**
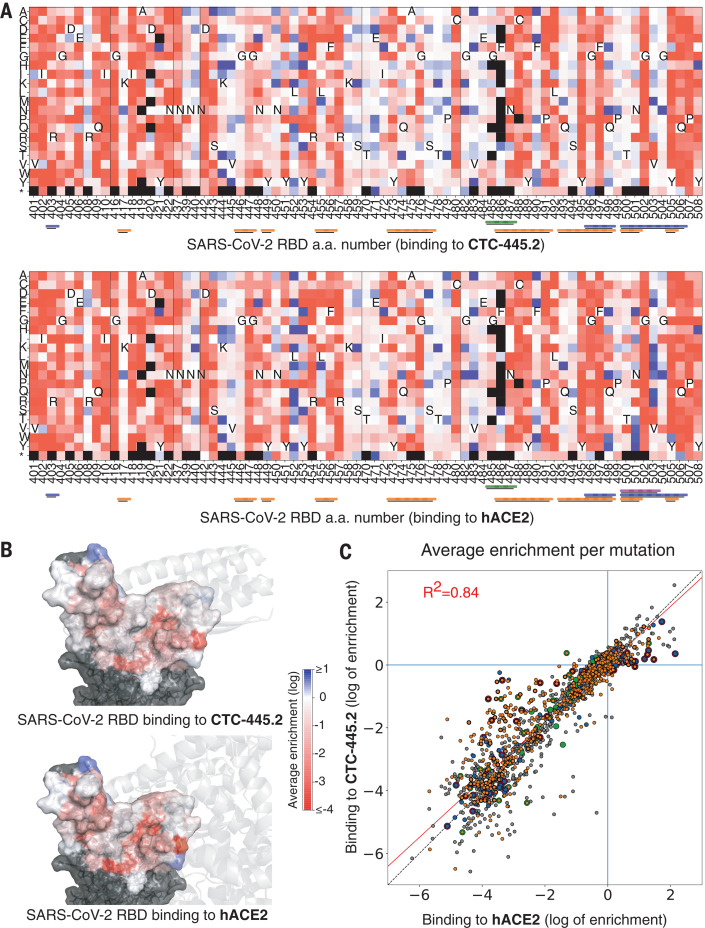
Resilience of CTC-445.2 to SARS-CoV-2 RBD mutational escape. (**A**) Deep mutational scanning (DMS) of the SARS-CoV-2 RBD interface was performed to assess the effect on binding (by yeast display) to CTC-445.2 (top) or hACE2 (bottom) at eight different concentrations (656, 218, 72, 24, 8, 2, 0.3, and 0.1 nM; fig. S16 and materials and methods). The heatmaps indicate the effect on binding for each possible single amino acid mutation in the hACE2-binding interface of the RBD (see the materials and methods). The results are the average over all the concentrations tested. A black square represents lack of expression in the naive (unselected) library. The color bars at the bottom indicate the secondary structure element with which a given RBD residue interacts: H1, orange; H2, green; EE3, blue; and H4, magenta. Approximately 1700 single mutations were targeted by the experiment. (**B**) The SARS-CoV-2 RBD surface is colored according to the per-residue-averaged enrichments for binding to CTC-445.2 (top) or hACE2 (bottom). For reference, the structure of CTC-445.2 or ACE2 (respectively) is shown in semitransparent gray cartoons. (**C**) The 2D scatter plots compare the enrichment values [as in (A)] for the DMS of the RBD binding to CTC-445.2 (*y*-axis) versus hACE2 (*x*-axis). There is a high correlation between the effect of RBD mutations in the binding of both molecules, demonstrating the mutational resilience of the de novo decoy (Pearson’s *r* = 0.92).

We also performed an SSM experiment for the SARS-CoV-2 RBD binding interface to compare the effect of single–amino acid substitution on binding to hACE2 or CTC-445.2. As predicted, the effects of ~1700 SARS-CoV-2 RBD mutations showed a strong correlation between binding to hACE2 and CTC-445.2 (*R*^2^ = 0.84, Pearson’s *r* = 0.92; Fig. 4 and fig. S14), highlighting the decoy’s intrinsic resiliency to mutational escape. At low target concentrations (100 pM), CTC-445.2 had a large binding advantage over ACE2 for many of the RBD mutations (fig. S14), likely a result of both its higher stability and smaller size. Although CTC-445.2 was resilient to viral mutations in the RBD-binding interface, we observed some decoy-binding–weakening mutations that had a lesser effect on hACE2 binding. Therefore, viral mutational escape might still be possible if multiple (decoy-binding–weakening) RBD mutations are combined.

**Fig. 5:**
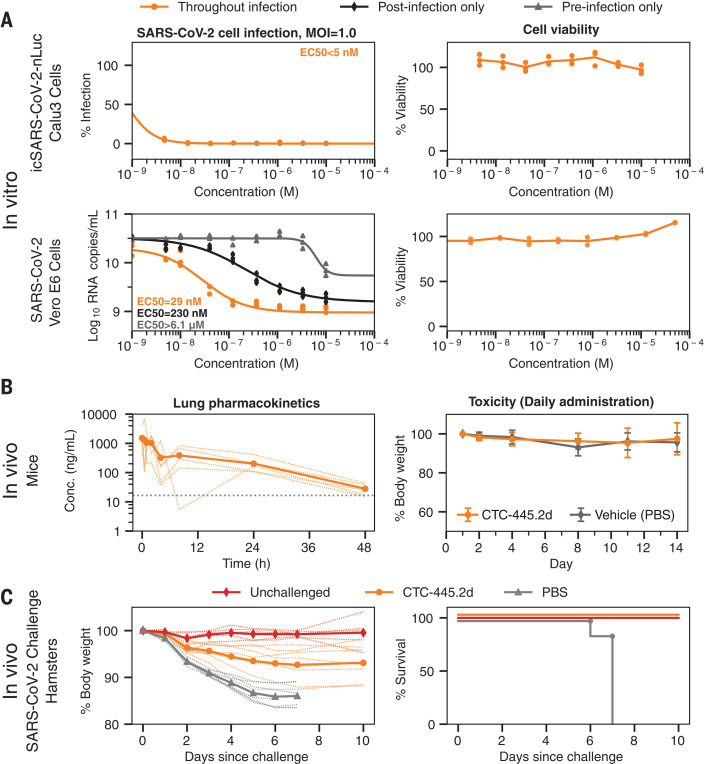
In vitro virus neutralization by CTC-445.2d. (**A**) Top left: In vitro neutralization of NanoLuc SARS-CoV-2 by CTC-445.2d in Calu-3 cells after 72 hours of incubation at a MOI of 1.0. Top right: A cell viability assay (48 hours) confirmed that the decoys are not cytotoxic to Calu-3. Bottom left: In vitro neutralization of live BetaCoV/Hong Kong/VM20001061/2020 SARS-CoV-2 virus in Vero E6 cells at a MOI of 1.0. The cells were incubated with CTC-445.2d throughout infection and the colors indicate the following: orange, before infection, during infection, and after infection; black, after infection only; and gray, before infection only. SARS-CoV-2 RNA copy numbers were determined by quantitative real-time reverse transcription polymerase chain reaction. All assays were performed in triplicate unless otherwise noted, and all data points are shown. Bottom right: Cell viability in Vero E6 cells was independently performed (CCK8 assay) and it was confirmed that the de novo decoys are not cytotoxic. (**B**) In vivo mouse pharmacokinetics and tolerability of intranasally administered CTC-445.2d. Left: Plot showing the concentration of fully functional CTC-445.2d (i.e., capable of binding to the SARS-CoV-2 RBD; see the materials and methods) found in homogenized lungs of Balb/c mice after a single 100 μg dose, measured at various times after dosing (*n* = 5 mice). Right: Body weight of mice after repeat daily intranasal doses of CTC-445.2d (100 μg; *n* = 18 at day 0) compared with control [phosphate-buffered saline (PBS)–treated] mice (*n* = 5). At each time point, three CTC-445.2d–treated mice were sacrificed for lung examination. Weight data shown are for the remaining mice (*n* = 18, 15, 12, 9, 6, and 3 at days 1, 2, 4, 8, 11, and 14, respectively). No significant weight loss or lung abnormalities were observed. Error bars indicate the standard deviation. (**C**) In vivo Syrian hamster SARS-CoV-2 challenge. Left: Body weight measurements through day 10 for unchallenged hamsters (*n* = 5, red) compared with SARS-CoV-2–challenged hamsters treated either with a single dose of CTC-445.2d (day 0 at –12 hours; *n* = 8, orange) or PBS (day –1, day 0 at –12 hours, day 1, and day 2; *n* = 7, gray). Right: Survival plot. Hamsters were euthanized when they displayed clinical signs of distress according to protocol clinical scoring criteria (see the materials and methods). At the end of the experiment, all hamsters treated with the de novo decoy CTC-445.2d survived, exhibiting moderate weight loss, whereas hamsters treated with vehicle did not survive past day 7 because of severe weight loss and other complications from the viral infection (see table S5).

The high and specific binding affinity of the optimized de novo protein decoys translated into effective and specific in vitro neutralization of SARS-CoV-2 viral infection (Fig. 5). In vitro, the presence of the de novo decoys had no impact on mammalian cell viability (Fig. 5A and fig. S15) or the enzymatic activity of hACE2 (fig. S16). Both of the decoys were able to fully neutralize viral infection in in vitro systems of cell infection. Briefly, in a vesicular stomatitis virus (VSV) pseudovirus system expressing the SARS-CoV-2 S protein, the decoys specifically protected human embryonic kidney (HEK) 293T cells overexpressing hACE2 from infection (fig. S15). The decoys also were able to fully neutralize infection by SARS-CoV-2 (SARS-CoV-2 nanoLuc; see the materials and methods) in the lung epithelial cell line Calu-3 expressing both ACE2 and the transmembrane protease serine 2 (TMPRSS2) (*35*, *36*) [median effective concentration < 5 nM at a multiplicity of infection (MOI) of 1.0; Fig. 5A]. In an in vitro time-of-addition assay using the Vero E6 cell line, CTC-445.2 and CTC-445.2d were most effective at neutralizing SARS-CoV-2 infection when continuously present in the cell media throughout the full course of infection (as opposed to only before or after infection; Fig. 5A and figs. S16 to S18), confirming that their mechanism of viral inhibition is extracellular neutralization of the virus.

To determine the potential of our molecules to be used as respiratory-delivered therapeutics, we intranasally administered a single dose of CTC-445.2d to Balb/c mice (100 μg dose of CTC-445.2d in a 30-μL droplet) and observed the presence of the fully functional decoy for >24 hours in the lungs and respiratory tract of mice (Fig. 5B and fig. S19). A 14-day course of daily CTC-445.2d intranasal administration in mice (100 μg of CTC-445.2d in a 30-μL droplet) was well tolerated, causing no adverse effects (Fig. 5B). In a Syrian hamster model for SARS-CoV-2 infection, a single prophylactic intranasal dose of CTC-445.2d (560 μg of CTC-445.2d in a 100-μL droplet) administered 12 hours before the viral challenge afforded 100% survival from a lethal SARS-CoV-2 challenge (5 × 10^5^ plaque-forming units of SARS-CoV-2; Fig. 5C). Specifically, by day 7, all control animals that received the viral challenge but not CTC-445.2d (*n* = 7) exhibited severe distress and required euthanasia. By contrast, hamsters that received a single dose of CTC-445.2d 12 hours before challenge all survived (*n* = 8), with modest weight loss and few or no clinical signs of distress (Fig. 5C and table S5).

Our de novo protein design approach to generate decoys is orthogonal to traditional therapeutics and has the potential to better overcome mutational viral evasion. Natural proteins repurposed often present substantial challenges for development as therapeutics; these include low stability, which can complicate manufacturing, transport, and storage; residual (and undesirable) biological activity; and the risk of eliciting an autoimmune response (*37*–*46*). By contrast, the de novo protein decoys are amenable for large-scale manufacturing in traditional bacterial systems, and their thermodynamic hyperstability can enable simplified transport and storage. Other recent protein-engineering efforts have generated neutralizing proteins characterized by extremely high binding affinities for SARS-CoV-2, with *K*_D_s ranging from low nanomolar to femtomolar [e.g., mAb 2B04 (*47*); LCB1 (*6*); and the nanobody Nb6 (*48*)]. Nevertheless, the de novo decoy’s resilience to viral escape is a distinctive feature of our design strategy (Fig. 4 and figs. S14 and S20). A possible shortcoming is that a decoy’s requirement to replicate a natural binding interface can intrinsically limit the maximum binding affinity attainable. However, we have demonstrated that the binding affinity (and potency) of the de novo decoys can be increased both by further sequence optimization (e.g., CTC-445.3d; fig. S21) or through avidity, allowing our trivalent decoy CTC-445.2t to reach the picomolar affinity range (Fig. 3I and fig. S10). It is possible that avid versions of CTC-445.2 coupled with more refined linkers (rigid and with proper spacing for binding simultaneously to multiple RBD subunits) might lead to larger increases in binding potency.

We demonstrate rapid design of a therapeutic lead; further speed improvements to our pipeline are theoretically attainable, for example by using high-throughput experiments to rapidly select and optimize the designs (Fig. 1G).

## Supplementary Materials

science.sciencemag.org/content/370/6521/1208/suppl/DC1 Materials and Methods Figs. S1 to S21 Tables S1 to S5 Appendix A: Python/PyRosetta code to generate multiple initial perturbations for mobile secondary structure elements References (*49*–*61*) MDAR Reproducibility Checklist
